# The genome as a record of environmental exposure

**DOI:** 10.1093/mutage/gev073

**Published:** 2015-10-06

**Authors:** Serena Nik-Zainal, Jill E. Kucab, Sandro Morganella, Dominik Glodzik, Ludmil B. Alexandrov, Volker M. Arlt, Annette Weninger, Monica Hollstein, Michael R. Stratton, David H. Phillips

**Affiliations:** Wellcome Trust Sanger Institute, Wellcome Trust Genome Campus, Hinxton, Cambridgeshire CB10 1SA, UK,; ^1^Analytical and Environmental Sciences Division, MRC-PHE Centre for Environment and Health, King’s College London, Franklin-Wilkins Building, London SE1 9NH, UK,; ^2^European Bioinformatics Institute (EMBL-EBI), Wellcome Trust Genome Campus, Hinxton, Cambridgeshire CB10 1SD, UK,; ^3^German Cancer Research Center (Deutsches Krebsforschungszentrum), Im Neuenheimer Feld 280, 69120 Heidelberg, Germany and; ^4^University of Leeds, Faculty of Medicine and Health, Leeds LS2 9JT, UK

## Abstract

Whole genome sequencing of human tumours has revealed distinct patterns of mutation that hint at the causative origins of cancer. Experimental investigations of the mutations and mutation spectra induced by environmental mutagens have traditionally focused on single genes. With the advent of faster cheaper sequencing platforms, it is now possible to assess mutation spectra in experimental models across the whole genome. As a proof of principle, we have examined the whole genome mutation profiles of mouse embryo fibroblasts immortalised following exposure to benzo[*a*]pyrene (BaP), ultraviolet light (UV) and aristolochic acid (AA). The results reveal that each mutagen induces a characteristic mutation signature: predominantly G→T mutations for BaP, C→T and CC→TT for UV and A→T for AA. The data are not only consistent with existing knowledge but also provide additional information at higher levels of genomic organisation. The approach holds promise for identifying agents responsible for mutations in human tumours and for shedding light on the aetiology of human cancer.

## Introduction

Mutagenesis drives the transformation of a normal cell to a tumour. A cancer genome is a historical record of mutagenic processes that have occurred throughout the life of a cancer patient, including mutations accrued during the normal part of the cell lineage as well as after neoplastic transformation (1–3). These mutations may result from endogenous mutagenic processes (e.g. spontaneous deamination of 5-methylcytosine) or exposure to exogenous mutagens, such as environmental chemicals or radiation. Additionally, mutations may develop due to failure of DNA repair pathways. A small number of mutations in a cancer cell (<10) are thought to confer a selective growth advantage (e.g. in *TP53* or other cancer genes) and are referred to as *driver* mutations (3). The vast majority of mutations in a cancer are simply bystander events, *passenger* mutations that report exclusively on the biological *mutational processes* that have occurred throughout cancer development (1–3). Each mutational process leaves its own characteristic mark or *mutational signature* on the cancer genome, defined by the mechanisms of DNA damage and DNA repair that constitute it (2,3).

A variety of experimental systems have been used to study the endogenous and exogenous factors driving mutagenesis. Traditionally, experimental mutagenesis studies have been limited to the analysis of mutations in a single gene (e.g., *HPRT*, *lacZ*, *cII*, *TP53*) which were identified in tumours or by specifically selecting for the growth of mutated cells or clones from mutagen-treated cell populations. Because each tumour or cell clone harboured only one or two mutations in a particular gene, patterns of mutations were inferred through pooling data collected from many samples, sometimes from different experiments.

The gene that is most commonly mutated in human cancers is the tumour suppressor gene *TP53* (4). Nearly 30000 *TP53* mutations identified in human tumours have been catalogued in the IARC TP53 mutation database (current version R17, http://p53.iarc.fr) and this resource has been valuable for identifying correlations between specific mutation signatures in human cancers and exposure to environmental mutagens [e.g. C>T and CC>TT mutations in squamous carcinomas of the head and neck, associated with ultraviolet (UV)-radiation exposure; G>T mutations in smokers’ lung cancer, associated with exposure to polycyclic aromatic hydrocarbons such as benzo[*a*]pyrene (BaP); A>T mutations in urothelial carcinomas, associated with exposure to aristolochic acid I (AAI)] (5). Some of these signatures have been recapitulated experimentally using cells from the partial human *TP53* knock-in (Hupki) mouse, in which exons 4–9 of human *TP53* replace the corresponding mouse exons (6,7). Immortalised clones derived from carcinogen-exposed primary Hupki mouse embryo fibroblasts (MEFs) harbour patterns of *TP53* mutation that closely resemble those identified in human tumours from patients exposed to the same carcinogens (8–11).

Although valuable insights have been gleaned from the study of single gene mutagenesis, such analyses cannot possibly illuminate all of the complex influences operating in the genomes of cancer cells. Not all human tumours have mutations in *TP53* and, of those that do, the mutation may be an early or late event in the pathogenesis of the tumour. Furthermore, a particular cancer sample usually has only one *TP53* mutation, thus mutational spectra must be obtained by aggregating mutations from many tumours, usually of the same type. This can be effective in reporting the signature of an exposure if there is a single dominant exposure in that cancer type, for example UV light in skin cancers or tobacco carcinogens in lung cancers. However, if multiple mutational processes have been operative in a particular cancer type, their signatures will become convoluted in the compiled *TP53* mutational spectrum. The observed *TP53* signatures may also be influenced by selection for particular driver mutations. Finally, it remains the case that signatures from only a small number of environmental carcinogens have been identified in human tumours from analysis of their *TP53* mutation patterns.

Massively parallel next-generation sequencing (NGS) technology (12) has resulted in an extraordinary increase in the speed and scale of sequencing, permitting the exploration of all protein-coding exons (exome sequencing) or whole genomes (whole-genome sequencing, WGS) in samples from patients or experimental model systems (13,14). This technology enables the detection of hundreds or even thousands of mutations in a single sample, increasing the power of each experiment considerably. Furthermore, the distribution of mutations throughout the genome can now be explored to gain further insights into mutagenic mechanisms. The complex biological insights buried within these large, multi-dimensional datasets can be dissected using mathematical separation approaches such as non-negative matrix factorisation (NNMF) (15). For example, NNMF has been used to extract at least 21 distinct mutation signatures from WGS data across 30 different cancer types (1) including a few signatures associated with exposure to carcinogens, such as tobacco smoke in lung cancer and UV radiation in malignant melanoma (1). Many novel signatures have also been uncovered (1) and the race is on to understand their aetiology.

In order to determine whether mutation signatures of *in vitro* carcinogen exposure could be extracted from a mammalian genome, and to explore additional insights that could be gained using a WGS approach, we sequenced the genomes of immortalised Hupki MEF clones exposed to the carcinogens BaP, AAI or ultraviolet C (UVC). In addition to determining the pattern and transcriptional strand bias for mutations induced by each agent, we were able to extract mutational signatures using NNMF and examine the effect of replication timing on mutagenesis. This study provides a perspective on how to use the genome as a physiological read-out of environmental exposures.

## Materials and methods

### MEF cell line information

The cell lines assessed here were derived in previous studies from primary Hupki MEF cultures exposed to the environmental carcinogens BaP, UV or AAI, as well as unexposed cultures (Supplementary Table 1). Briefly, primary MEFs were isolated from Day 13.5 Trp53^tm/Holl^ mouse embryos which contain the Hupki gene, exposed to BaP (1 µM, 6 days), AAI (50 µM, 4 days), UVC (20 J/m^2^), or left untreated, and then subjected to serial passaging according to a modified 3T3 protocol (16,17). Immortal clones that emerged from senescent cultures were passaged at low density for several passages to establish cell lines. The lines chosen for this study each harboured an inactivating *TP53* mutation (Supplementary Table 1). Genomic DNA was isolated from the cells by a standard phenol/chloroform extraction method and stored at −20°C.

### Massively parallel sequencing and alignment

Short insert 500bp genomic libraries were constructed, flowcells prepared and sequencing clusters generated according to Illumina library protocols (18). 100 base paired-end sequencing was performed on Hiseq 2000 genome analyzers in accordance with the Illumina Genome Analyzer operating manual. Short insert paired-end reads were aligned to the reference mouse genome (NCBIM37) using Burrows-Wheeler Aligner, BWA (v0.5.9) (19).

### Processing of genomic data

#### Mutation-calling: substitutions

A bespoke algorithm, CaVEMan (Cancer Variants Through Expectation Maximisation: http://cancerit.github.io/CaVEMan/) was used for calling somatic substitutions in MEFs. CaVEMan utilises an expectation-maximisation (EM) algorithm to call variants in second-generation sequencing reads. Given the reference base and copy number status, CaVEMan generates a probability score for potential genotypes at each genomic position. A high specificity was essential for the purposes of downstream analyses. As such, further postprocessing filters of potential somatic genotypes were designed to eliminate false positive calls arising from: genomic features that generate mapping errors, for example regions of excessively high coverage due to collapsed repeat sequences in the reference genome (http://genome.ucsc.edu/), systematic sequencing artifacts, for example motifs known to cause errors of phasing during the sequencing reaction or sequencing artifacts arising in at least 5% of reads in at least 2 samples from a panel of unrelated samples, misalignment caused by germline insertions/deletions and germline SNPs.

#### Mutation-calling: insertions/deletions

Insertions and deletions in the immortalised and primary MEF genomes were called using a modified Pindel version 2.0. on the mouse NCBIM37 genome build (20). Variants were screened against a panel of unrelated samples. Indels were further filtered against possible germline SNPs (dbSNP).

#### Mutation-calling: structural variation

Structural variants were first discovered using a bespoke algorithm, BRASS (BReakpoint AnalySiS) (https://github.com/cancerit/BRASS) through discordantly mapping paired-end reads from short insert data by using BWA alignments. Next, discordantly mapping read pairs that were likely to span breakpoints, as well as a selection of nearby properly paired reads, were grouped for each region of interest. Using the Velvet *de novo* assembler (21), reads were locally assembled within each of these regions to produce a contiguous consensus sequence of each region.

### Transcriptional strand characterisation

The nucleotide sequence of the primary mRNA transcript is identical to the sense/non-template/non-transcribed strand except that U replaces T, and is complementary to that of the anti-sense/template/transcribed strand. All mutations were called on the + strand of the reference genome, were placed into the ‘pyrimidine’ context and noted if so. Transcriptional strand was assigned for each pyrimidine-based mutation. Assignment of transcriptional strands was performed by considering only protein coding genes. Of the total of 22556 protein coding genes, 11209 (513086613bp) were on the + strand and 11347 (494818895bp) were on the − strand.

### Replication domain characterisation

Reference coordinates for replication landmarks were inferred from Repli-seq data obtained from the ENCODE project (22) (https://www.encodeproject.org/) for MEFs. In Repli-Seq experiments, cell lines are first isolated into cell cycle fractions of newly replicated DNA and each fraction is sequenced. To visualise genome-wide replication patterns as a continuous function, percentage-normalisation of sequencing tags was followed by a wavelet-smoothed transformation. The resolution of these data is relatively limited when compared to the data obtained from Repli-Seq experiments performed in humans. Original data contained 384796 probes (average size of 61bp) encompassing 234727739bp of the mouse reference genome. ENCODE blacklist for mm9 genome (22) was used to exclude regions showing an artificially high signal. This filtering process removed 280 probes, and a final list of 384516 probes encompassing 23455659bp of the mouse reference was obtained.

#### Replication time domains

For analyses requiring conservative distinctions between early and late replication time domains, an EM algorithm for multivariate normal mixtures was applied. The model was based on the assumption that the observed Repli-seq signal was a random sample from a finite normal mixture of three distributions (corresponding to early, late and unknown domains) (Supplementary Figure 1A), where each distribution can be completely described by its mean *μ*_*i*_ and variance $\sigma_{i}^{2}$. Standard EM algorithm for normal mixtures was used to estimate the parameters *μ*_*i*_ and variance $\sigma_{i}^{2}$ associated with the three normal distributions fitting the Repli-seq data. The intersections between the fitted normal distributions were used to classify the domains. Adjacent probes belonging to the same replication time domain were merged to obtain larger regions. The final list contained 1270 early replicated regions (average size of 523813.8bp for a total of 676673544bp) and 1458 late replicated regions (average size of 772977.4bp for a total of 1127001075bp). All analyses were corrected for genomic size of the relevant regions. AT/GC content (Supplementary Figure 1B) and distribution of protein-coding genes (Supplementary Figure 1C) are in-keeping with expectations for early and late replication domains.

#### Characterisation of replication strands

Replication origins do not fire as a part of a clear, deterministic program, instead occurring individually and as clusters (23–25). Replication strands were defined using Repli-seq signals: peaks (local maxima) in the smoothened profile correspond to replication initiation zones while valleys (local minima) correspond to replication termination zones (22). Finite difference approximations of second and first derivatives were used to identify Repli-seq signal local maxima [*f*″(*x*) < 0] and local minima [*f*″(*x*) > 0] corresponding to potential origin firing sites, and to distinguish between leading [*f*′(*x*) < 0] and lagging [*f*′(*x*) > 0] strand, respectively. Derivative functions were defined in agreement with p and q arm chromosome orientation. We named the replication strand as p2q-leading and p2q-lagging. As for replication time domains, adjacent probes belonging to the same replication strand were merged to obtain larger regions. In order to remain conservative in downstream assignments (26,27), we only considered merged regions containing at least three probes, discarding ambiguous regions that were <10kb in length. The final list contained 11568 p2q-leading regions (average size of 103452.6bp for a total of 1196739303bp) and 11579 p2q-lagging regions (average size of 101,406.1bp for a total of 1174180686bp). Derived p2q-leading and p2q-lagging strands are comparable in genomic footprint and AT/CG content (Supplementary Figure 1D). However, there were no differences observed in the distribution of mutations between leading and lagging strands, respectively.

## Results

### Whole genome mutation spectra and strand bias in carcinogen-exposed MEFs

**Figure 1.**
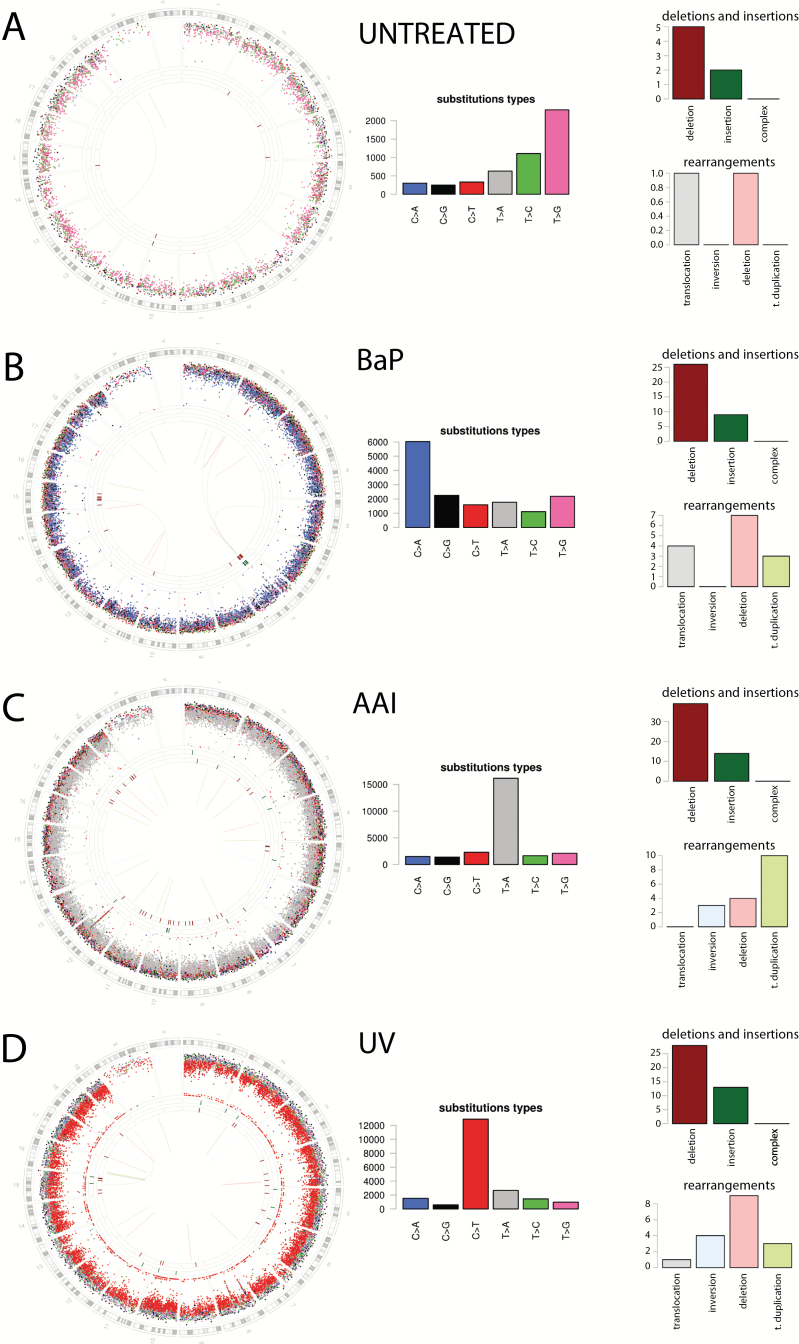
Whole-genome sequenced read-outs of four different Hupki MEF clones exposed to different mutagens demonstrate distinctive differences between clones. (**A**) from a clone of untreated cells, containing 4913 mutations; (**B**) from a clone of BaP-treated cells, containing 14929 mutations; (**C**) from a clone of AAI-treated cells, containing 25100 mutations; (**D**) from a clone of UV-irradiated cells, containing 20111 mutations. Features in Circos plots on left depict karyotypic ideogram outermost, then moving inwards: base substitutions, plotted as rainfall plots (log10 intermutation distance, dot colours: blue = C>A, black = C>G, red = C>T, grey = T>A, green = T>C, pink = T>G). Ring with short green lines = insertions, ring with short red lines = deletions. Central line: grey = intrachromosomal rearrangements, pink = deletions, green = tandem duplications. Histograms in the centre show substitution types. Histograms on right show deletions and insertions (upper) and rearrangements (lower). The *y*-axis of each histogram indicates the number of mutations. Enlarged versions of the Circos plots can be viewed in Supplementary Figures 2–5.

Following WGS we identified 14929, 25100 and 20111 mutations, respectively, in each treated subclone (BaP, AAI and UV). In addition, 4913 mutations were identified in the untreated subclone. Exploring the totality of mutations in these cell lines (Figure 1), striking visually discernible differences can be appreciated by the substitution mutational spectra while also demonstrating a lack of a specific phenotype in the indels and rearrangements in these otherwise structurally quiescent genomes.

The mutation spectra identified in these subclones were typical of those previously reported to be associated with each of the exposures. Hupki MEFs treated with BaP had substantial numbers of C>A (substitutions are referred to by the pyrimidine of the mutated Watson-Crick base pair) transversions, characteristic of bulky DNA adducts formed at guanine residues by the major reactive metabolite of BaP, benzo[*a*]pyrene-7,8-diol-9,10-epoxide. AAI-treated MEFs showed a majority of T>A transversions, a mutational pattern characteristic of bulky adducts formed at adenine residues by reactive aristolactam nitrenium ions following AAI metabolism. C>T transition mutations were the most common type of mutation seen in MEFs treated with UV radiation and a significant proportion of these were highly distinctive CC>TT double substitutions, typical of mutagenesis associated with pyrimidine dimers caused by UV exposure. Intriguingly, untreated MEFs carried largely T>G transversions.

**Figure 2.**
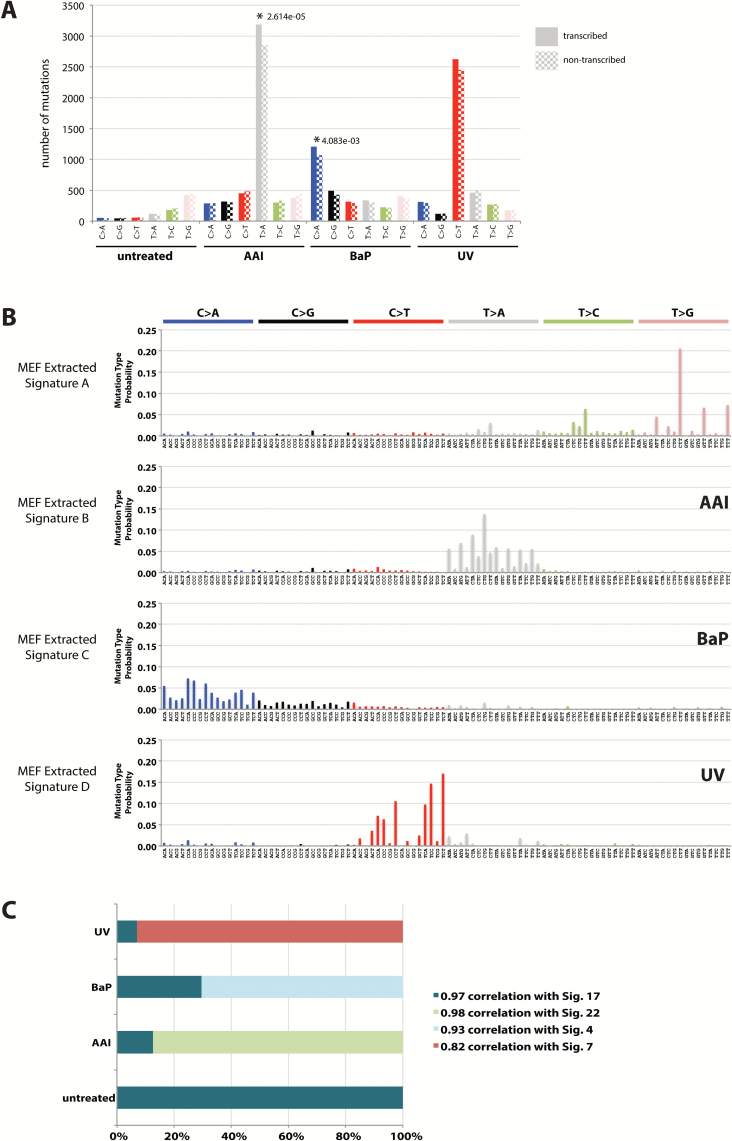
(**A**) Transcriptional strand bias of somatic base substitutions derived from Hupki MEFs exposed to various mutagens. Blue = C>A, black = C>G, red = C>T, grey = T>A, green = T>C and pink = T>G. *P*-value for significance calculated using a binomial proportions test. (**B**) Mutation signatures extracted from base substitutions in treated and untreated MEFs. (**C**) The proportion of mutations attributed to human cancer signatures in each MEF cell line. Pearson correlation for similarity of MEF extracted signatures to those previously extracted from primary human cancers is provided. An enlarged version of B can be viewed in Supplementary Figure 6.

We found that a transcriptional strand bias was detectable in cells treated with AAI or BaP, with a significant excess of mutations found on the non-transcribed strand for A>T transversions in the AAI-treated MEFs and for G>T transversions in the BaP-treated MEFs (Figure 2A). This is in keeping with the activity of transcription-coupled nucleotide excision repair (TC-NER) on the transcribed strand, a branch of NER that removes RNA polymerase II-blocking DNA lesions caused by each of these mutagens. Mutations in MEFs treated with UV, however, did not exhibit a transcriptional strand bias. This is in contrast to what has been observed in UV-associated human tumours, where C>T mutations are biased to the non-transcribed strand (1). The reason for this difference is unclear, but perhaps the repair of UVC-induced DNA damage in MEFs is different to that of sunlight-induced (i.e. UVA/UVB) DNA damage in human cells.

### Mutation signatures extracted using NNMF

The mathematical method NNMF allows extraction of complex mutation signatures of 96 elements (six base substitution classes with four 5′ and four 3′ flanking base options). This algorithm and its application is described in detail by Alexandrov *et al.* (15) Performing NNMF on the WGS profiles from the untreated and mutagen-treated cell lines, we found four distinct mutation signatures (Figure 2B). Three were unique to individually treated MEFs and corresponded to known environmental exposures (Figure 2C): the signature identified in MEFs treated with BaP (Extracted Signature C) was similar to Signature 4, which was extracted previously from smoking-associated lung cancers; the signature identified in MEFs treated with UVC radiation (Extracted Signature D) was similar to Signature 7 extracted from skin cancers associated with UV exposure; the signature identified in AAI-treated MEFs (Extracted Signature B) was nearly identical to Signature 22 extracted from urothelial cancers caused by exposure to the associated mutagen (http://cancer.sanger.ac.uk/cosmic/signatures). Curiously, however, one signature was present in all four MEF clones, including the cells that had not been exposed to a mutagen. This signature (Extracted Signature A) is identical to Signature 17 and has been identified primarily in stomach and oesophageal cancers, hepatocellular carcinoma and lymphoma. This very distinctive signature has been reported in a variety of different cell types including *in vitro* mouse cell models (daughter subclones of organoids derived from murine gastrointestinal tract cells) (28). The cause of this signature, however, remains unknown.

NNMF also permits quantification of the amount of the different signatures in each of the cell clones. We found that each of the signatures associated with specific carcinogen exposures was identified only in individually treated MEFs, apart from Signature 17, which was present in all four MEF lines whether treated or not. This implies that a background mutational process causing Signature 17 was acting in all the MEF lines.

### Relationship of mutation spectra to genomic architecture

WGS approaches permit the exploration of relationships between the patterns of mutagenesis and features of the genome. Exploiting resources such as ENCODE, the genome can be compartmentalised according to replication time and strands based on RepliSeq experiments previously performed on MEFs. Although we did not find any significant differences in the distribution of mutations between leading and lagging replicative strands for base substitutions (data not shown), we observed intriguing differences in the distribution of mutations between early and late replication domains (Supplementary Figure 7). With the exception of C>T transitions in the UV-treated MEFs, the dominant mutation class for each of the treated MEFs (T>A in AAI-treated and C>A in BaP-treated) and untreated MEFs (T>G mutations) demonstrated a higher mutation density late in replication, regardless of whether they were within the gene footprints (transcribed vs. non-transcribed) or intergenic. The unusual behaviour of C>T mutations in the UVC-treated MEFs could be due to the fact that the cells were acutely exposed to UVC only once, whereas cells were exposed to BaP and AAI for several days and DNA damage would have occurred throughout several rounds of replication. It remains to be determined whether chronic exposure to UV light would alter the distribution of mutations compared with a single, acute treatment. These analyses provide first insights into how to capitalise on analyses of genome-wide mutagenesis.

## Discussion

From this proof-of-principle study, several important principles emerge. WGS of MEFs treated with mutagens reveals patterns of mutation previously observed in single gene analyses (e.g. *TP53*) but with much larger numbers of mutations, allowing greater precision of characterisation of the mutational signature than could ever have been obtained from analysis of a single gene. Strikingly, this was achieved by sequencing only a single sample for each mutagen exposure. Even subtle differences in mutational signature, for example between different components of tobacco smoke, may in future be detectable given these amounts of data. Transcriptional strand bias and the impact of many other features of the genome landscape upon mutagenesis are extractable with the statistical power available from such studies. Other studies coming to similar conclusions using exome sequencing have recently been published (29–31). However, the extraordinary number of mutations and the much greater variation in genome landscape features visible from a whole genome sequence will undoubtedly provide great additional richness of insight.

*In vitro* model systems (e.g. cell-based) provide a means for studying patterns of mutagenesis under controlled conditions, now at the level of the genome. This can provide a detailed mutation signature to connect with a specific mutagenic exposure that far exceeds current understanding. To reduce costs associated with sequencing or to increase experimental power, model organisms with smaller genomes such as *Saccharomyces cerevisiae* (12 Mbp), *S. pombe* (12.57 Mbp) and *Caenorhabditis elegans* (100Mb) may, in some instances, be tractable systems to sequence compared with the mouse (2.5 Gb) or human genome (3.2 Gb). Multiple clones can be sequenced at a relatively modest cost. As a comparison, using current sequencing techniques, one lane of a HiSeq 2000 Illumina sequencing run provides ~10-fold coverage of a human genome or an equivalent sequence coverage of 100 yeast clones. Recently, Meier *et al.* (32) examined whole genome mutation signatures in *C. elegans* exposed to the mycotoxin aflatoxin B1 and two chemotherapeutic agents, cisplatin and mechlorethamine. Mutation profiles induced by these carcinogens reflected the known biochemistry of each agent and resembled signatures observed in human cancers and developmental genomic disorders, thus indicating that such an approach could be applied for additional carcinogens. However, differences in genome composition, genome architecture, cellular physiology and DNA repair pathways exist between species and the generalisability of sequencing approaches from model organism to humans may be limited. Furthermore, smaller (non-human) genomes are smaller targets for mutation, such that many more genomes (and hence many more libraries for sequencing) may be required to obtain the same amount of information that is obtainable from larger (human or mouse) genomes, eliminating any potential cost reductions.

As we attempt to identify the possible environmental origins of mutation signatures observed in human tumours, it would be useful to have a curated dataset of signatures extracted from human cells exposed to specific mutagens under controlled conditions. The advent of newer, faster genome sequencing technologies means that we can move towards this goal, whereby mutations can be examined across the exome or the whole genome, with or without a selection procedure. Key to the successful documentation of such data is a well-constructed experiment. Here, a proposition would be to use normal human cells as a reference parental line, such as human induced pluripotent stem cells, which are indefinitely proliferative and easily subcloned. Cells could be treated with mutagens suspected of causing cancer in humans, and daughter subclones, preferably isolated and expanded without any phenotypic selection processes, could be derived and used for identifying genome-wide mutational signatures. Ideally such cells should have the capacity to metabolically activate mutagens, but, if not, exogenous metabolising systems (e.g. S9 mix) could be incorporated into the mutagen-treatment protocol. In theory, each daughter cell line should carry the signature associated with the specific mutagen exposure. Background mutagenesis associated with culture or other shared exposures will be detectable and quantifiable (and therefore easily subtracted). These curated signatures of known cause could then be included in a reference database, which could then be compared with signatures extracted from human cancers, normal human cells or other sources to look for similarities that might provide clues to cancer aetiology.

Based on analyses of single gene mutation spectra, only a handful of carcinogens have yet been shown to induce unique mutational fingerprints, whereas many agents generate overlapping spectra (33). It is axiomatic that a wealth of additional information will emerge from widening the scope of analysis from a single gene to the whole genome, whereby WGS will likely reveal more complex spectra that are unique to individual mutagens. Further, carcinogen-induced mutation signatures can be expanded to include a wider sequence context, dinucleotide base substitutions and insertions/deletions. This approach will be highly useful in elucidating the endogenous and exogenous origins of mutations in human cells and in identifying the causative agents of human cancers. With many cancers suspected of being influenced by as-yet-unidentified environmental causes, the tracking down of these causes will be a crucial step towards achieving the ultimate public health goal of cancer prevention.

## Supplementary data

Supplementary Table 1 and Figures 1–7 are available at *Mutagenesis* Online.

## Funding

Research was funded in part by Cancer Research UK (C313/A14329) and the Wellcome Trust (101126/Z/13/Z and 101026/B/13/Z). S.N.-Z., J.E.K., V.M.A., M.R.S. and D.H.P. are members of the Wellcome Trust funded COMSIG (Causes of Mutational SIGnatures) consortium. S.N.-Z. is personally funded by a Wellcome Trust Intermediate Clinical Research Fellowship (WT100183MA) and is a Wellcome-Beit Fellow.

## Supplementary Material

Supplementary Data
