# Supplementary material for: The genome as a record of environmental exposure
Source: Mutagenesis. 2015 Oct 6;30(6):763–70. doi: 10.1093/mutage/gev073 (PMC4637815; doi:10.1093/mutage/gev073)
Supplement: Supplementary Data [file supp_30_6_763__index.html]

The genome as a record of environmental exposure — Supplementary Data 

# The genome as a record of environmental exposure

## Supplementary Data

Data files

- Supplementary Data - Supplementary Data
- Supplementary Data - Supplementary Data
- Supplementary Data - Supplementary Data
