# Supplementary material for: The genome as a record of environmental exposure
Source: Mutagenesis. 2015 Oct 6;30(6):763–70. doi: 10.1093/mutage/gev073 (PMC4637815; doi:10.1093/mutage/gev073)
Supplement: Supplementary Data [file supp_gev073_Supplementary_Figure_Legends.docx]

**Supplementary Figure Legends**

1. A) Overview of the approach used to extract the conservative early and late replication domains, where normal distributions were fitted to the MEF Repli-seq signal using a multivariate normal mixture approach. Intersections between the curves were used to define conservative replication domains. B) Relative proportions of nucleotides for replication time domains. C) Genomic size (base pairs on y axis) dedicated to protein-coding genes in early and late replicating domains obtained from the MEF line from ENCODE. D) Comparison of genomic size (base pairs on y axis) dedicated to each of the nucleotides (A,C,G,T and N) between leading and lagging replicative strands.
2. Enlarged Circos plot from Figure 1A (untreated MEFs).
3. Enlarged Circos plot from Figure 1B (BaP-treated MEFs).
4. Enlarged Circos plot from Figure 1C (AAI-treated MEFs).
5. Enlarged Circos plot from Figure 1D (UV-treated MEFs).
6. Enlarged MEF Extracted Signatures from Figure 2B.
7. Mutation densities (y-axis) in different genomic replication domains (early versus late, x-axis) is provided for aggregated mutations in each MEF cell line, for mutations separated by substitution classes (C>A, C>G, C>T, T>A, T>C and T>G) and also separated by whether they were intergenic (diamonds), transcribed (triangles) or non-transcribed (squares) strands.
