# Supplementary material for: The genome as a record of environmental exposure
Source: Mutagenesis. 2015 Oct 6;30(6):763–70. doi: 10.1093/mutage/gev073 (PMC4637815; doi:10.1093/mutage/gev073)

Supplementary Figure 1

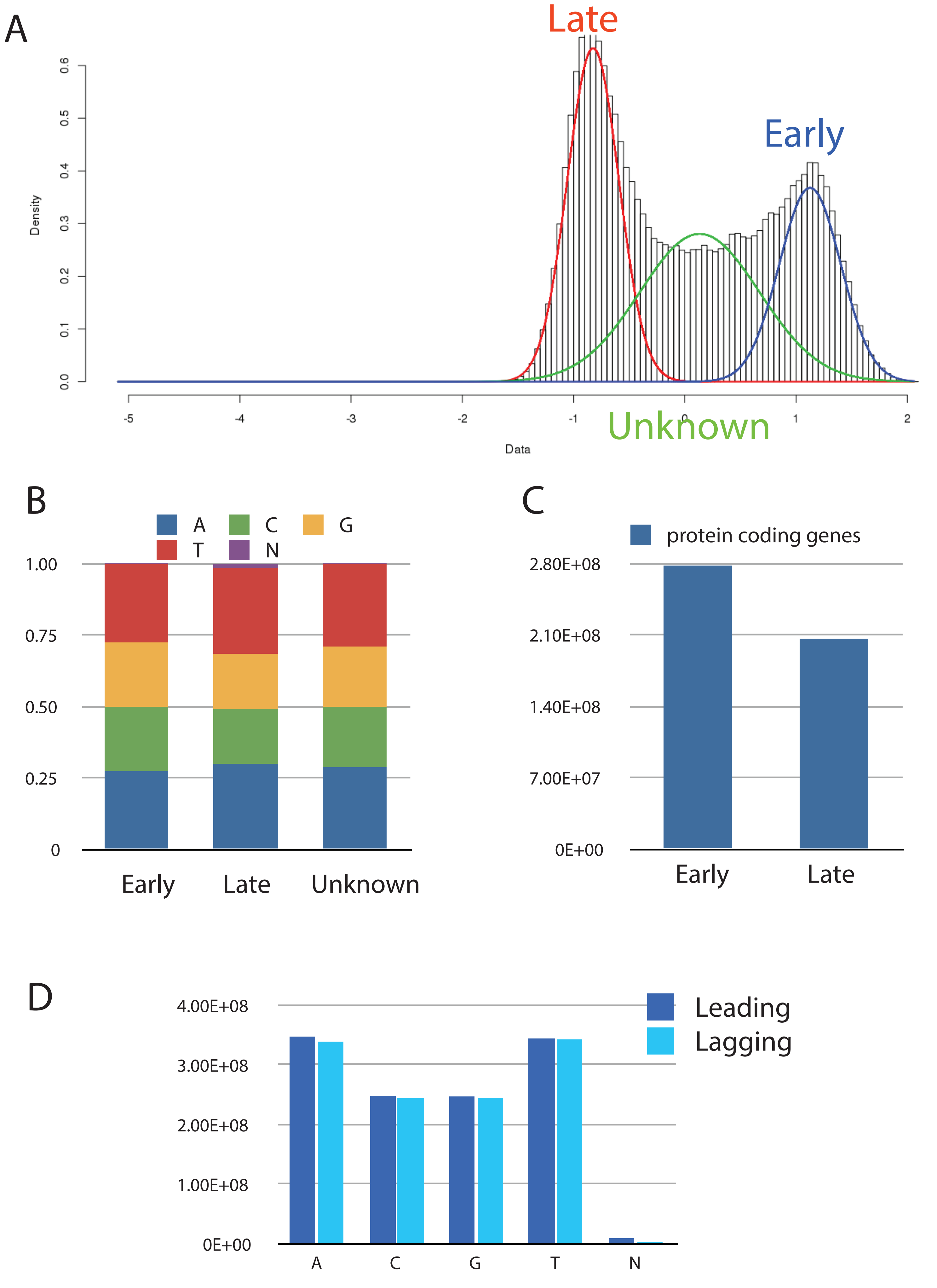

Supplementary Figure 2: UNTREATED

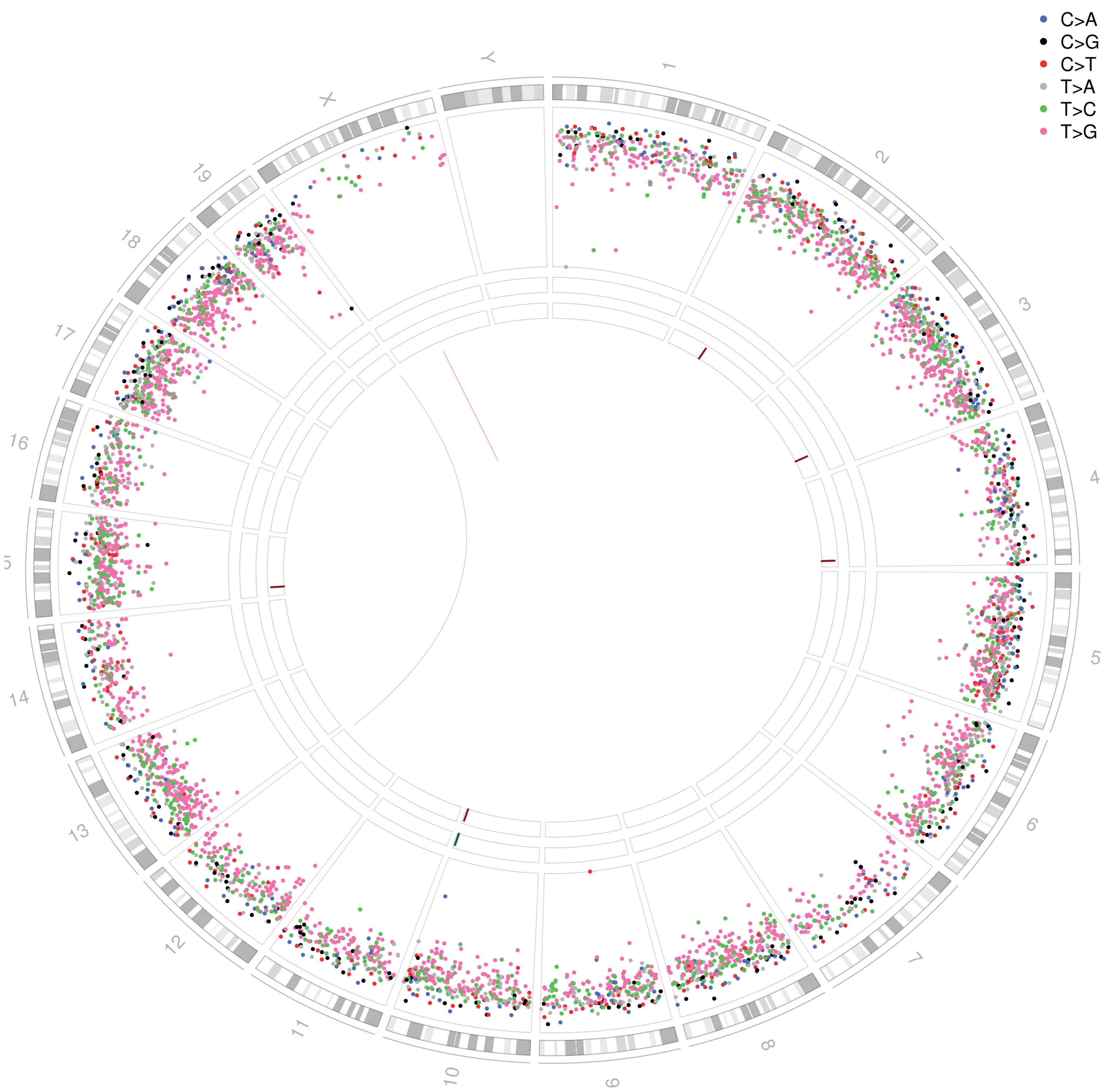

Supplementary Figure 3: BaP

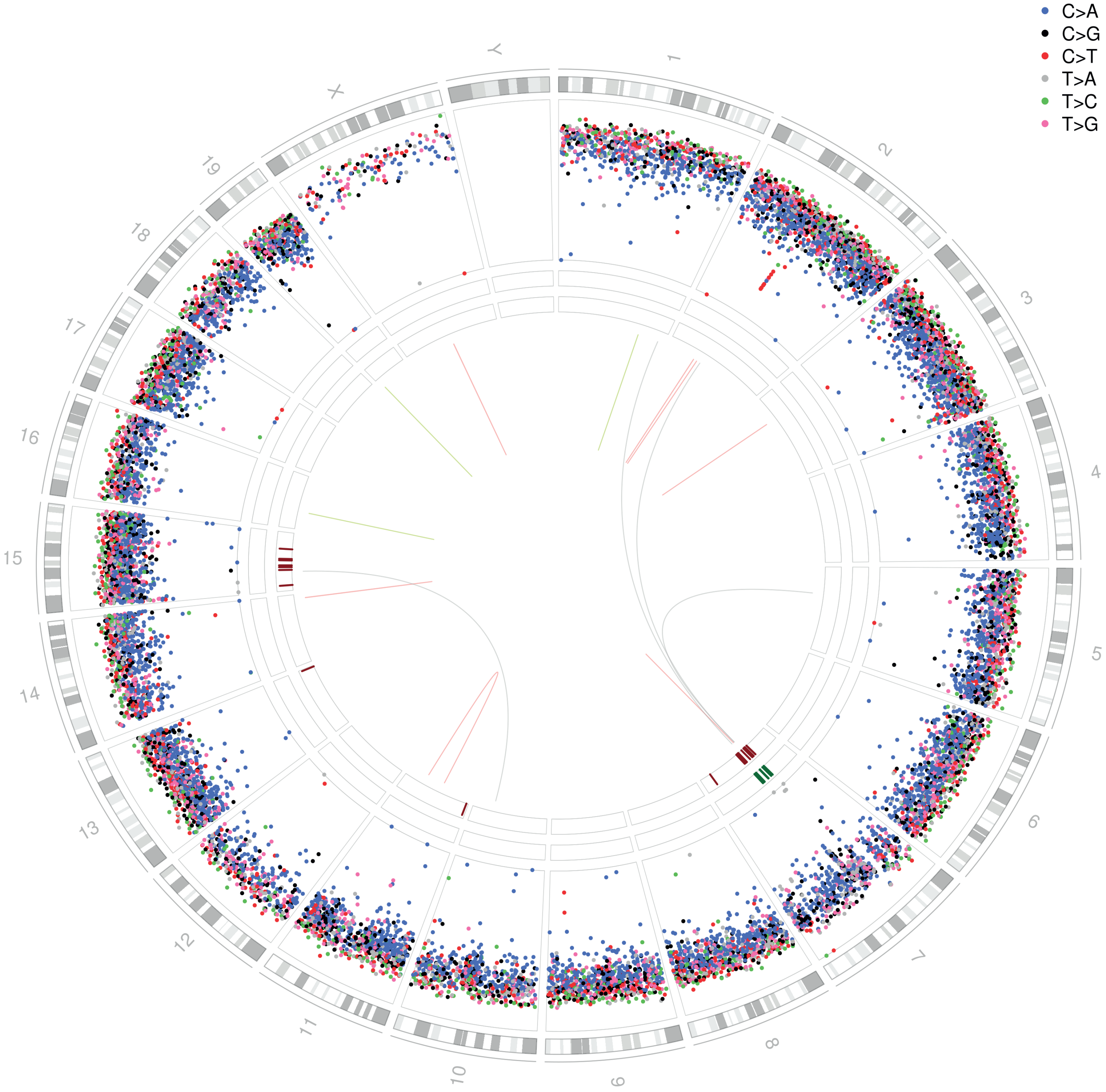

Supplementary Figure 4: AAI

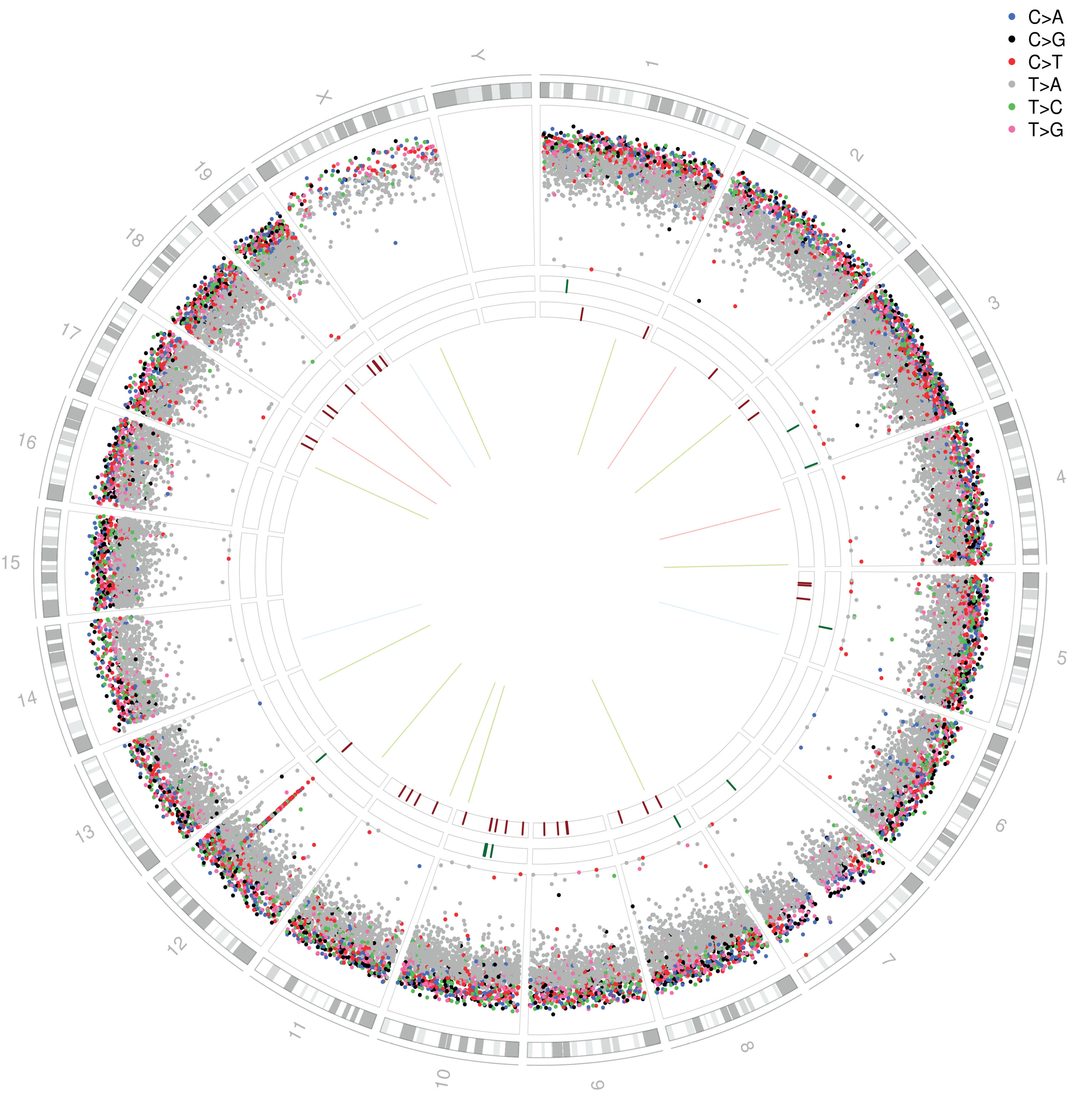

Supplementary Figure 5: UV

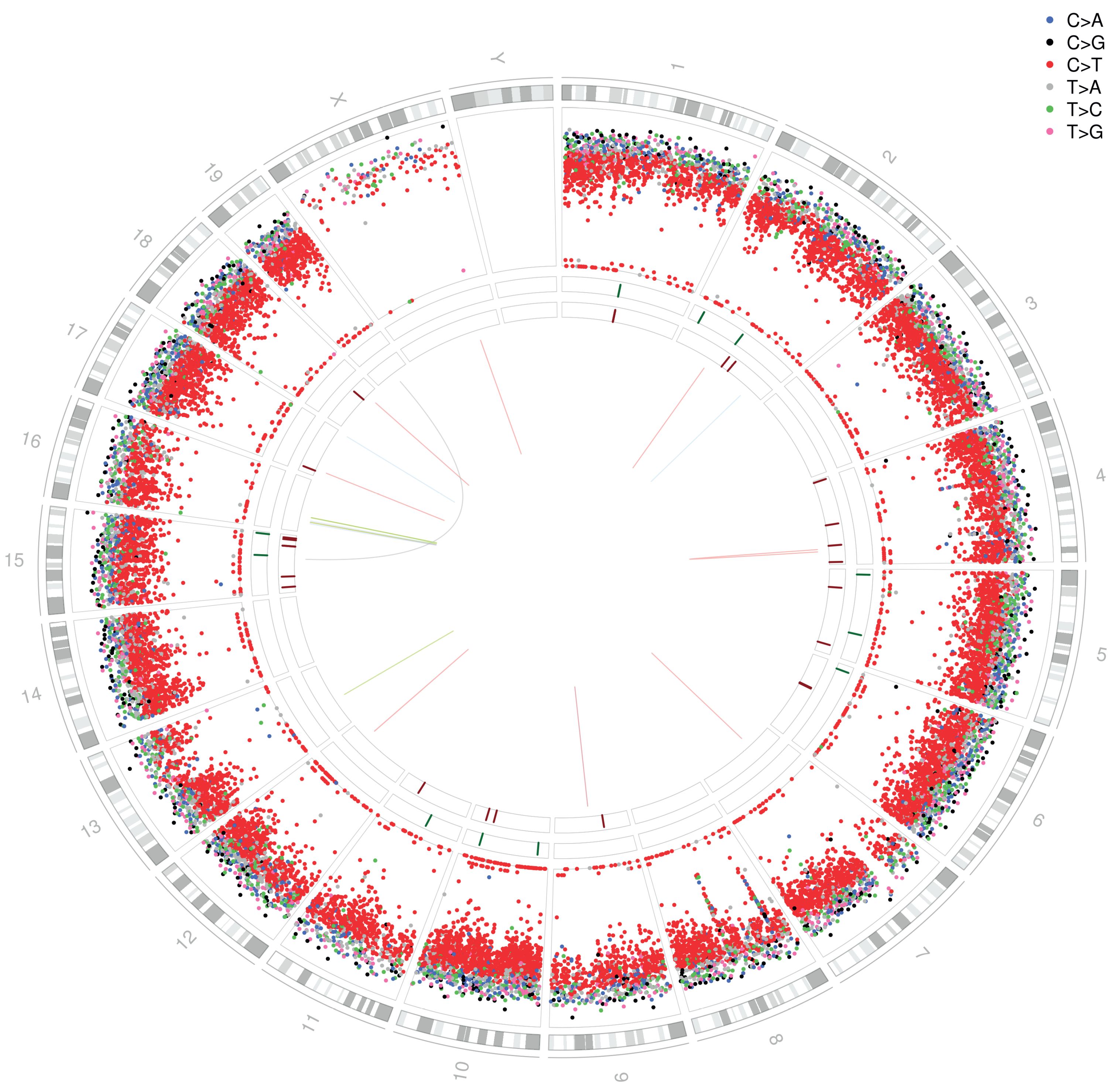

Supplementary Figure 6

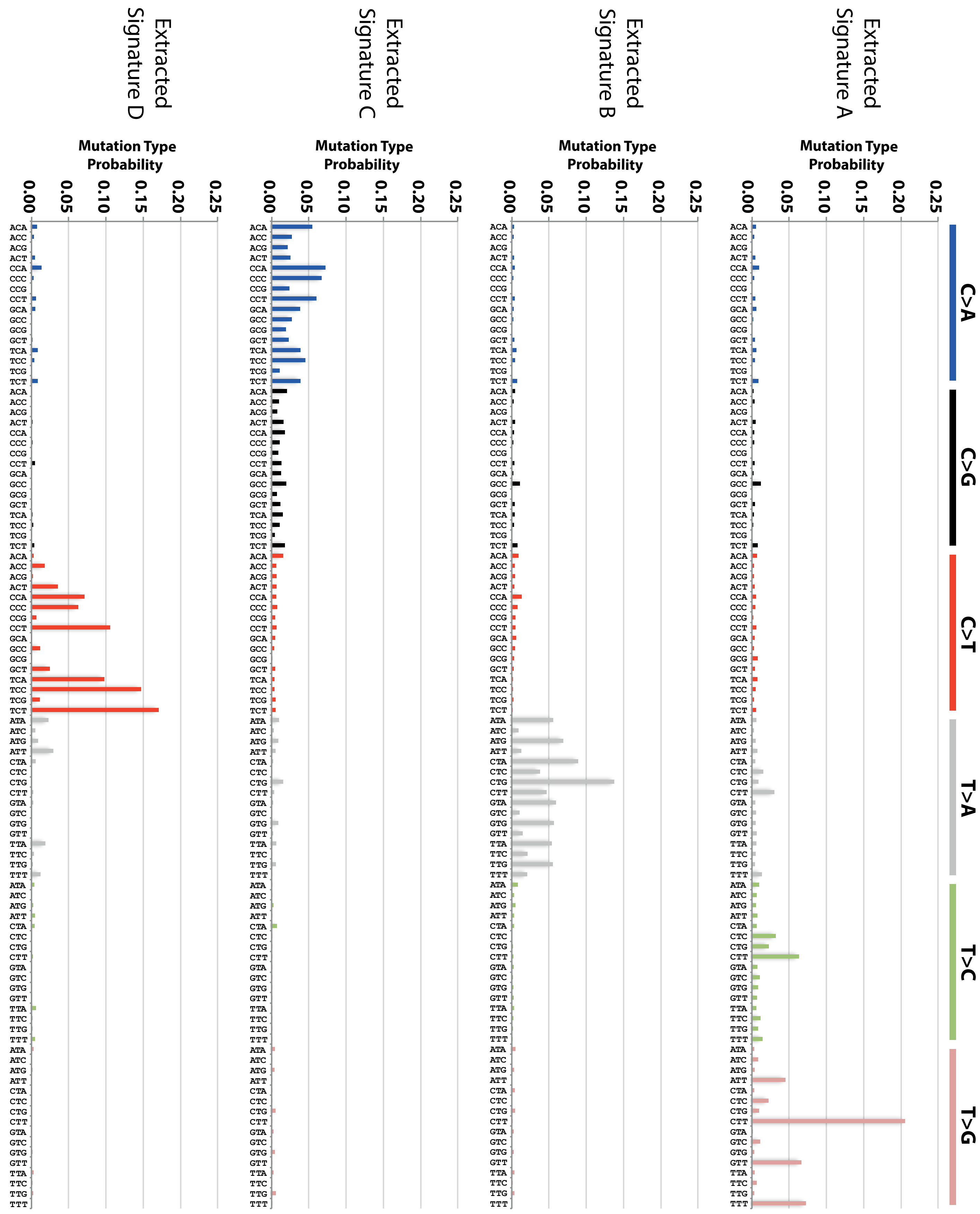

Supplementary Figure 7

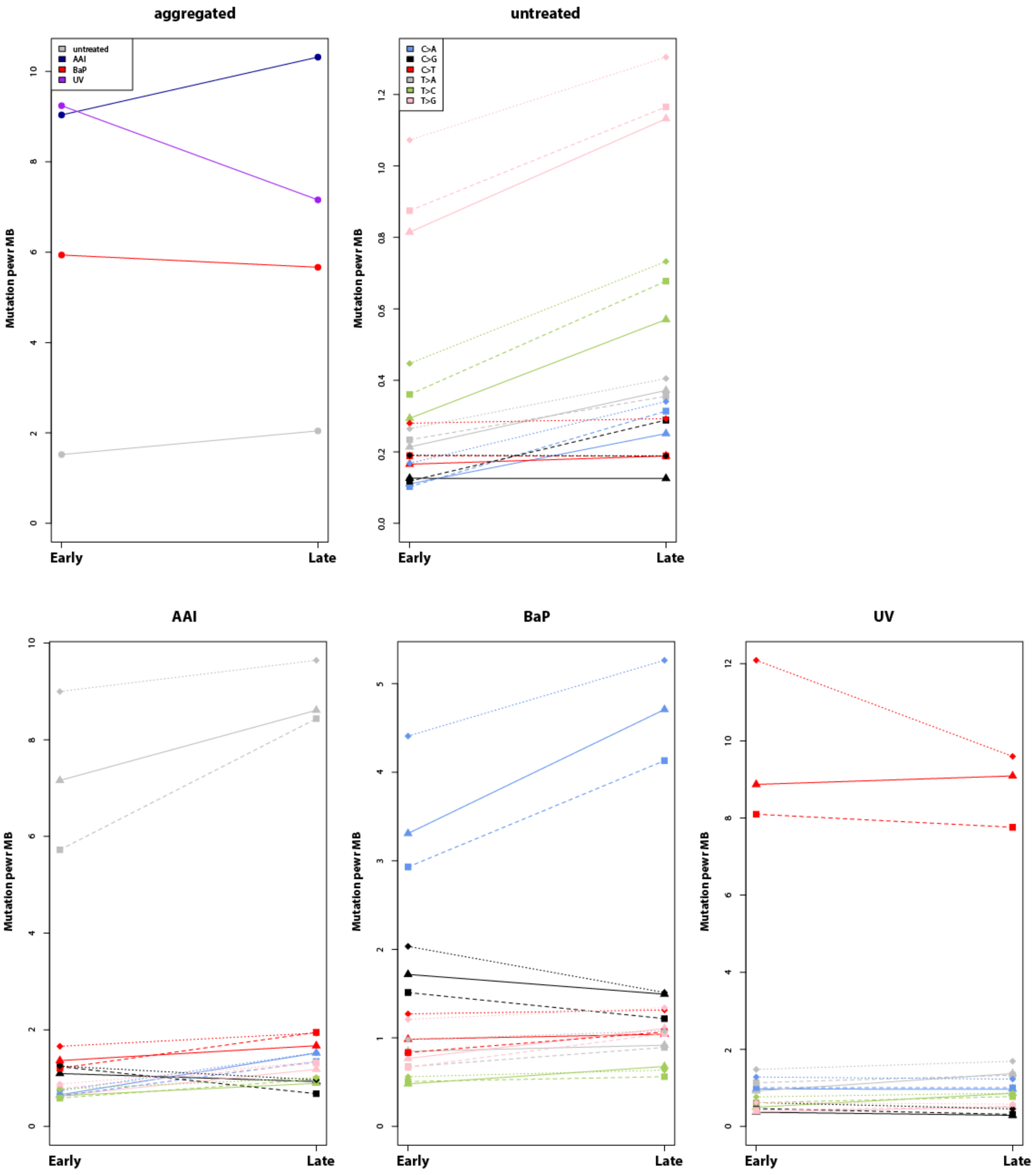

Supplement: Supplementary Data [file supp_gev073_Supplementary_Figures_1_7.pdf]
